# “Pain, Stress, and Emotions”: Uncontrolled trial of a single-session, telehealth, emotional awareness and expression therapy class for patients with chronic pain

**DOI:** 10.3389/fpain.2022.1028561

**Published:** 2022-11-18

**Authors:** Maisa S. Ziadni, John A. Sturgeon, Mark A. Lumley

**Affiliations:** ^1^ Department of Anesthesiology, Perioperative and Pain Medicine, Stanford University School of Medicine, Palo Alto, CA, United States; ^2^ Chronic Pain and Fatigue Research Center, Department of Anesthesiology, University of Michigan School of Medicine, Ann Arbor, MI, United States; ^3^ Department of Psychology, Wayne State University, Detroit, MI, United States

**Keywords:** EAET, chronic pain, single-session, telehealth, pain intensity, pain interference, pain catastrophizing

## Abstract

**Objectives:**

Trauma- and emotion-focused chronic pain interventions, particularly Emotional Awareness and Expression Therapy (EAET), show much promise for reducing pain and improving functioning. We developed a novel, single-session, telehealth-delivered EAET class (“Pain, Stress, and Emotions”; PSE) and tested it on adults with chronic pain of mixed etiology.

**Methods:**

After an initial developmental phase, we conducted an uncontrolled trial, providing PSE to 74 individuals with chronic pain (63.5% female; 64.9% White; 60.8% with pain duration >5 years) in four class administrations. Participants completed self-report measures (primary outcomes: pain intensity and pain interference) at baseline and multiple follow-ups to 12 weeks. Linear mixed-models examined changes over time, and effect sizes were calculated on change from baseline to 4-week (primary endpoint) and 12-week follow-ups. The trial was registered with clinicaltrials.gov (NCT05014126)

**Results:**

Participants reported high satisfaction with the PSE class. Pain intensity showed a significant, medium reduction across time (*p* < .001; *d *= 0.60 at 4 weeks); one-quarter of participants had clinically meaningful pain reduction (≥30%). Pain interference had a large reduction (*p* < .001; *d *= 0.74). There were significant but smaller improvements in most secondary outcomes (*ds = *0.15 to 0.55; *ps* < .01). Effects were generally maintained or increased at 12-week follow-up. Higher education and baseline ambivalence over emotional expression predicted greater pain reductions.

**Conclusions:**

People taking this EAET class had reduced pain severity and interference and improvements in other pain-related outcomes. The single-session, telehealth class holds promise as an easily delivered, efficient, and potentially impactful intervention for some patients with chronic pain, although controlled trials are needed.

## Introduction

Chronic pain remains difficult to treat. Various psychological or behavioral interventions, including cognitive-behavioral, acceptance, and mindfulness-based therapies, have been found to have positive effects on pain-related outcomes such as pain intensity, interference, and distress. These therapies, however, have two limitations—magnitude of effect and accessibility.

Several meta-analytic reviews indicate that the effects of these leading treatments for chronic pain are limited in magnitude, with generally small benefits for pain reduction and only slightly larger effects on pain interference (1–3). It has been proposed that these somewhat limited benefits may be due, in part, to the failure of these therapies to directly address the trauma, interpersonal stressors, psychological conflicts, and emotional problems that are found in many patients with chronic pain and that appear to trigger, amplify, or maintain pain (4, 5). A growing body of evidence from trauma- and emotion-focused interventions for chronic pain suggests the possibility of enhanced effects (6–8). In particular, Emotional Awareness and Expression Therapy (EAET) was developed specifically to target such underlying emotional drivers of pain, especially primary, nociplastic, or centralized pain. This therapy emphasizes pain neuroscience (the brain as amplifying or even generating pain), disclosure of emotionally salient traumas or conflicts, and active processing and resolution of trauma/conflict through expressive writing, in-session expression exercises, and direct communication in relationships. Uncontrolled studies of EAET suggest substantial effects on pain and somatic symptom reduction (9, 10), and two randomized trials of 8-session in-person, group EAET found it to be superior to Cognitive Behavioral Therapy (CBT) on pain reduction in older military veterans (11), and on some secondary pain outcomes in people with fibromyalgia (12).

Accessibility is a limitation of all current therapies, including EAET. A key accessibility barrier is requiring patients to attend multiple sessions in person, which is how these therapies are routinely conducted in both clinical trials and in practice. The time, travel, and cost of in-person, multi-session therapies, along with limited availability in remote or rural areas and a lack of adequately trained clinicians to reach such patients, leads not only to a lack of access for many patients in need, but also increased attrition for those who can participate (13). The COVID-19 pandemic has further challenged the feasibility of multisession, in-person treatments (14). There clearly is a need for remotely-administered, brief, group-based interventions to have greater reach and potentially greater population impact (15).

In response to this need, a single-session, remotely delivered class that teaches patients cognitive-behavioral skills (specifically targeting pain catastrophizing) has been developed. This “Empowered Relief” course has been found effective in several trials (16–18). A single-session version of EAET—delivered in-person and to individuals rather than groups—was developed and shown to improve pain-related outcomes in a study of primary care patients (19) and women with urogenital pain (20). To date, however, there is no single-session version of EAET that is delivered remotely or to groups of patients. Such a class, especially one that asks patients to engage in emotionally difficult experiences (acknowledge / disclose adversities, express avoided emotions) might be hard to conduct (i.e., lack feasibility), rejected by patients, or even psychologically upsetting. These concerns, however, need to be tested empirically.

To address these issues, we develop a novel, single-session, telehealth-delivered EAET class, which we labeled for patients “Pain, Stress, and Emotions” (PSE). After initial development and preliminary testing, we then conducted an uncontrolled trial of PSE, offering multiple classes for people with chronic pain. We had several goals. First, we sought to examine the effectiveness of this intervention as measured by changes from baseline in participants' pain intensity and pain interference (primary outcomes) and multiple secondary outcomes at 4-week (primary endpoint) and 12-week (secondary endpoint) follow-ups. Second, we explored several baseline variables as individual difference predictors of the effects of this PSE class. Specifically, we hypothesized that higher levels of baseline adverse childhood experiences or ambivalence over emotional expression—and lower levels of emotional approach coping—would predict better outcomes of PSE, because EAET targets those with adversity and who avoid emotional engagement and expression. Third, to address concerns about how an EAET class would be received by patients, we examined patient's reactions to the class, including acceptability, satisfaction, and comprehension.

## Materials and methods

### Study design and oversight

This uncontrolled, prospective trial was coordinated by Stanford University School of Medicine and had class leaders and patients from both the local area and various states. The study protocol was approved by the Stanford University Institutional Review Board (IRB), and electronic consent was obtained from all participants. We utilized an iterative development approach in which we first conducted an initial pilot class with 11 participants, after which we improved procedures and finalized study slides and protocols for the uncontrolled trial. The trial was then registered with clinicaltrials.gov (NCT05014126), and patients were enrolled—and classes conducted—between August 2021 and December 2021, with follow-up assessments completed in February 2022. The study followed the consolidated Standards of Reporting Trials (21) reporting guidelines on clinical trials.

### Patient participants

We recruited adults with chronic pain of mixed etiology who had agreed to be contacted for research studies. Inclusion criteria were: (1) having pain of at least 3 months' duration; (2) aged 18–80 years; and (3) ability and willingness to complete electronic questionnaires and to participate in the class *via* a Zoom platform during a scheduled time. We excluded people who had ongoing legal action related to pain or a disability claim or who had cognitive impairment, serious psychiatric disorders, or were non-English speaking.

### Procedure and assessments

Interested participants completed an online screening form, and those who were eligible were contacted by study staff and enrolled. Participants provided electronic informed consent and were informed of several dates/times that the PSE class would be offered over the upcoming weeks. Seven days prior to the class that they planned to attend, patients were emailed a link to baseline questionnaires, along with information on when and how to attend the class (Zoom link, etc.) The baseline assessment included demographic variables (age, sex, race, ethnicity, relationship status, education, occupation, and annual household income) and measures of outcomes and potential predictors, as listed below. One day after the class, a brief treatment satisfaction and acceptability survey was administered electronically. Participants then completed the same baseline battery of measures electronically (minus demographics) at four follow-up times: 2, 4, 8, and 12 weeks after the class. The primary study endpoint was the 4-week follow-up, and the secondary endpoint was the 12-week follow-up; these two time-points are the focus of data presentation. The additional follow-up assessments (2-week and 8-week) were collected to allow us to more closely track change trajectories and to provide more data for multilinear modeling. Participants were compensated up to $100 for completing all of the assessments; the class was provided at no charge.

### Study intervention: “Pain, Stress, & Emotions” class

We developed a single-session, 2-hour, Zoom-delivered, EAET class for chronic pain. Each class was led by two doctoral-level psychologists with expertise in EAET. The class was delivered with a Powerpoint presentation but was also interactive and experiential. The first hour focused on: (a) pain psychology and neuroscience education, including the role of the brain in pain; (b) the limitations of imaging for identifying bodily sources of pain; (c) the model of chronic pain as often being a “false danger alarm” that can be triggered not only by fear of injury or pain but also of other psychosocial threats; and d) how to “teach the brain safety by reversing avoidance of fear-inducing experiences. We also presented four brief exercises from Kohns et al. (2020) (22) to help patients examine the role that stress and emotions play in their pain and to personalize their learning: (a) various central sensitization conditions/symptoms they have had, (b) personality traits or emotional needs that create anxiety, (c) life stressors that often trigger or exacerbated pain, and (d) the 10-item Adverse Childhood Experiences Scale. Participants responded to these exercises by placing answers in the chat or holding up fingers or hands; that is, sharing their answers with group members and leaders. The first hour of the class ended with an experiential exercise in which each patient practiced communicating language reflecting assertion / anger and connection/vulnerability (with microphones muted) to an empty chair, and their reactions (anxiety, guilt) were discussed.

For the second hour, participants were assigned randomly to one of two break-out rooms (*n* = 8–10 participants in each), and each break-out room led by one of the two instructors. These smaller groups allowed more interaction with individual patients. We first presented a model of interpersonal stress that drives pain and the role of emotional expression as the key to reversing the trauma and pain. For the next 8–10 min, all participants then engaged in a brief expressive writing exercise at home—writing an unsent letter about a personal trauma or conflict—expressing both connecting and angry/assertive feelings. Next, patients were invited to verbally disclose/share with the break-out group some part of their story / writing, and typically two or three patients did so. Next, we invited one patient to engage in an expression in which the group leader helped the patient communicate verbally and expressively to the “other person,” who was imagined to be next to them in the room. The leader helped the patient find language and express both sets of feelings more explicitly and intensely to the other person than the patient might have done in their writing. During the entire second hour, patients were asked to track and report their pain and their anxiety, and leaders commented on how changes were linked with emotional experiences. The class ended with both breakout groups rejoining as a larger group for about 10 min and discussing their observations and what they had learned. Finally, participants were encouraged to set individual goals by completing a personalized prescription plan for promoting their emotional, relational, and physical health.

### Primary outcomes

#### Pain intensity

Respondents rated their average pain intensity over the previous 7 days on a numerical pain rating scale (NPRS) of 0 (*no pain*) to 10 (*worst pain imaginable*) (23). Assessment of pain intensity using an NPRS has been supported in prior studies (24).

#### Pain interference

The NIH Patient-Reported Outcomes Measurement Information System (PROMIS) short-form measures have been applied in pain research (25–33), and selected domains were identified by the Initiative on Methods, Measurement, and Pain Assessment in Clinical Trials (IMMPACT) (34, 35) as core outcomes. Respondents reference the previous 7 days to rate items. The short form of pain interference (6a) was utilized to assess self-reported consequences of pain on different aspects of a person's life including engagement with social, cognitive, emotional, physical, and recreational activities. Raw total scores on this and other PROMIS short-form measures were converted to *t*-scores using conversion tables available on the PROMIS Assessment Center website (http://assessmentcenter.net). Higher scores indicate higher interference with activities. Internal consistency of the pain interference measure was high (*α* = .91).

### Secondary outcomes

Patients completed the PROMIS measures for sleep disturbance (version 6a), physical function (version 8b), depression (version 6a), anxiety (version 6a), anger (5a), fatigue (6a), and social isolation (6a). Higher scores on PROMIS measures indicate greater symptom severity, except for physical function, wherein higher scores reflect better function. In addition, patients completed the13-item Pain Catastrophizing Scale (PCS) (36) which measures patterns of negative cognition and emotion in the context of actual or anticipated pain. The response scale ranges from 0 (*not at all*) to 4 (*all the time*); total sum scores range from 0 to 52. Internal consistency was high for all PROMIS short forms (*α* ranging from.90 to.94). The PCS has good internal psychometric properties (37), and the internal consistency of the PCS in our sample at baseline was high (*α* = .94).

### Exploratory predictors of intervention response

#### Ambivalence over emotional expression

The 14-item version of the Ambivalence over Emotional Expression Questionnaire (AEQ) (38, 39) assesses participants’ ambivalence or conflict over the external expression of one's feelings (e.g., “Often I’d like to show others how I feel, but something seems to hold me back”). Items were rated from 1 (*I have never felt like this*) to 5 (*I feel like this a lot*) and averaged; higher scores indicate greater ambivalence over emotional expression. In our sample, the scale had high internal consistency at baseline (*α* = .90).

#### Emotional approach coping

The 8-item Emotional Approach Coping (EAC) scale (40) assesses both emotional processing and emotional expression (41). Patients rated items on a 4-point scale from 1 (*I don't do this at all*) to 4 (*I do this a lot*), and ratings were summed to yield a total EAC score (ranging from 8 to 32). Higher scores indicate greater levels of emotional approach coping. In our sample, we found high internal consistency for the total scale (*α* = .80).

#### Adverse childhood experiences

The 10-item Adverse Childhood Experiences (ACEs) scale (42) assesses several types of adverse childhood experiences, including neglect, abuse, poverty, and parental conflict or substance abuse, that occurred during the first 18 years of life. The ACE includes yes or no questions, and each “*yes*” response receives one point, for a total score that ranges from 0 to 10. ACEs has an adequate test/retest reliability over 1 year (*r* = 0.64, *p *< .001) (42).

### Feasibility and acceptability of intervention

The assessment of feasibility and acceptability of the PSE zoom-delivered intervention replicated published methods (16). We obtained participant ratings of treatment acceptability, satisfaction, relevance, usefulness of information presented, ease of understanding, and likelihood to use the skills learned. The rating scale ranged from 0 to 10; with higher scores indicating higher acceptability and satisfaction across respective items. We also asked participants for open text responses regarding general feedback on the class and what they found to be particularly helpful or challenging about the class.

### Statistical analyses

We included analyses for all patients who took the class, including those missing outcome data, using linear mixed modeling from SPSS 28.0 to test for significant changes in each primary and secondary outcome across all 5 timepoints (baseline and 4 post-class assessments). Linear mixed modeling is appropriate for use in statistical models using time series or hierarchical data and is robust in cases of missing data. Random effects were specified for individual intercepts (means) and slopes were specified as fixed given the relatively small sample size. To determine the size of the intervention effect at planned endpoints, we calculated Cohen's *d* by computing change scores from baseline to a set time point, divided by the baseline standard deviation. These computations were carried out for comparisons from baseline to 4 weeks (primary endpoint) and from baseline to 12 weeks (secondary endpoint). As an exploratory step, we conducted an analysis of baseline variables (pain duration, disability status, and education), and three potential predictors of response that were identified *a priori* (AEQ, EAC, and ACEs). Separate models were estimated testing an interaction between baseline scores of each potential predictor and lower-order effects predicting primary outcomes (pain intensity and pain interference). Below is an example multilevel model demonstrating a cross-level interaction predicting differential changes in an outcome across time according to baseline levels of ambivalence regarding emotional expression:

$$Y_{ij}=\beta_{0j}+\beta_{1j}*\mathrm{Time}+\beta_{2j}*\mathrm{AE}Q_{ij}+\beta_{3}*\mathrm{Tim}e_{ij}*\mathrm{AE}Q_{ij}+e_{ij}$$

For the models examining potential predictors of treatment response, all time points (i.e., assessments at baseline and 2-, 4-, 8-, and 12-week follow-ups) were included to better represent the full trajectory of treatment responses during the study period. Any significant interactions were subsequently depicted graphically with participants grouped according to low (1 standard deviation below sample mean), medium (sample mean) and high (1 standard deviation above sample mean) scores on the predictor.

## Results

### Sample characteristics

Sample characteristics of the 74 patients in the trial are presented in Table 1. The mean age of the sample was 50.4 years (SD = 15.4, range 21–79); 47 participants (63.5% of the sample) identified as female, 24 as male (32.4%), and 3 as non-binary (4.1%). About two-thirds of the sample (*n* = 48, 64.9%) was White, with a range of other races/ethnicities. Median education was a completed associate's degree, and median income was $105,000–$124,999 per year. Half were married or living with a partner, and just over half reported full- or part-time employment.

**Table 1:** Baseline sample characteristics (*N* = 74).

Variable	*n* (%)
Age Mean (SD)	
50.4 (15.4)	Range: 21–79
Gender	
Female	47 (63.5%)
Male	24 (32.4%)
Nonbinary	3 (4.1%)
Hispanic ethnicity	12 (16.2%)
Racial identity	
Caucasian	48 (64.9%)
Asian	13 (17.6%)
Other/Unknown	6 (8.2%)
More than one race	3 (4.1%)
American Indian/Alaska Native	2 (2.7%)
Native Hawaiian or Pacific Islander	1 (1.4%)
Black/African American	1 (1.4%)
Education level	
High School or GED	2 (2.7%)
Some college, no degree	7 (9.5%)
Associate's or Vocational Degree	10 (13.5%)
Bachelor's Degree	34 (45.9%)
Master's Degree	16 (21.6%)
Professional School Degree	2 (2.7%)
Doctoral Degree	3 (4.1%)
Income	
Less than $10,000	2 (2.7%)
$10,000 to $24,999	6 (8.1%)
$25,000–$44,999	7 (9.5%)
$45,000–$64,999	4 (5.4%)
$65,000–$84,999	8 (10.8%)
$85,000–104,999	11 (14.9%)
$105,000–124,999	5 (6.8%)
More than $125,000	31 (41.9%)
Marital Status	
Married	37 (50.0%)
Never married	20 (27.0%)
Divorced	8 (10.8%)
Partnered, Living together	3 (4.1%)
Widowed	3 (4.1%)
In relationship, not living together	2 (2.7%)
Separated	1 (1.4%)
Disabled	
No	70 (94.6%)
Yes	4 (5.4%)
Employment Status	
Full time	29 (39.2%)
Retired	16 (21.6%)
Unemployed	13 (17.6%)
Part time	10 (13.5%)
Disabled	7 (9.5%)
Homemaker	7 (9.5%)
Pain Duration	
3–6 months	2 (2.7%)
6–12 months	1 (1.4%)
1–5 years	26 (39.2%)
More than 5 years	45 (60.8%)

Regarding pain characteristics, mean pain intensity for the sample were 4.95 (SD = 1.66, range 1–10) at baseline. A majority of the sample reported pain duration greater than 5 years (*n* = 45, 60.8% of the sample), 26 participants reported pain duration between 1 and 5 years (35.1%), and 3 participants reported pain for 3–12 months (4.1%). Pain diagnosis or pain type was assessed *via* self-report, and participants could endorse more than one category; the most commonly-endorsed was low back pain (67.6%), but neck pain (48.6%), general musculoskeletal pain (29.7%), head pain (non-migraine; 28.4%), and arthritis (28.4%) were also commonly reported. Full self-reported pain diagnosis data can be found in Table 2.

**Table 2:** Pain diagnoses of the trial sample (*N* = 74).

Pain diagnosis	*N* (%)
CLBP	50 (67.6%)
Neck pain	36 (48.6%)
Musculoskeletal pain	22 (29.7%)
Arthritis	21 (28.4%)
Headaches- non-migraine	21 (28.4%)
Degenerative disc disease	18 (24.3%)
Migraine	15 (20.3%)
Other	14 (18.9%)
Myofascial/muscle pain	10 (13.5%)
Temporomandibular joint dysfunction	10 (13.5%)
IBS	9 (12.2%)
Carpal tunnel	8 (10.8%)
Neuropathic pain	8 (10.8%)
Fibromyalgia	7 (9.5%)
Post-surgical/surgery recovery	7 (9.5%)
Pelvic Pain	6 (8.1%)
CRPS/RSD	5 (6.8%)
Abdominal pain	5 (6.8%)
Trigeminal neuralgia	2 (2.7%)

### Study flow

In this uncontrolled trial, the PSE class was offered four times, with 17 to 19 participants in each class. Regarding follow-up assessments, 70 participants (94.6% of the sample) were assessed at 2 weeks, 68 (91.9%) at 4 weeks, 69 (93.2%) at 8 weeks, and 70 (94.6%) at 12 weeks. A total of 72 participants (97.3%) provided at least one follow-up assessment (only 2 had no follow-up data), and 65 participants (87.8%) had complete data at all time points.

### Primary outcomes

Descriptive statistics, including effect sizes for primary and secondary outcomes at the primary and secondary endpoints, are presented in Table 3. LMM models indicated a significant reduction in average pain intensity across the study period (*B *= −.15, *p *= .002). At 4-week follow-up, 17 out of the responding 68 participants (25%) reported at least 30% reductions in pain intensity, 9 of whom (13.2%) reported pain intensity reductions of 50% or greater. At 12 weeks, 20 out of 70 participants (28.6%) reported at least 30% pain reduction, 8 of whom (11.4%) reported pain intensity reductions 50% or greater. Effect size calculations suggested a medium magnitude reduction in pain intensity from baseline to 4 weeks and a small-to-medium reduction from baseline to 12 weeks.

**Table 3:** Primary and secondary outcomes at baseline and primary and secondary endpoints (4- and 12-week follow-ups).

Outcome	Baseline M (SD) (*n* = 74)	4-week Follow-up M (SD) (*n* = 68)	Effect size (*d):* Baseline to 4 weeks	12-week follow-up M (SD) (*n* = 70)	Effect size (*d):* Baseline to 12 weeks
Primary					
Pain intensity	4.95 (1.66)	3.96 (1.92)	.60	4.26 (2.09)	.42
Pain interference	63.0 (5.52)	58.9 (8.09)	.74	59.0 (8.00)	.72
Secondary					
Pain catastrophizing	20.3 (11.21)	14.1 (10.44)	.55	13.3 (10.11)	.63
Depressive symptoms	55.5 (8.72)	53.3 (9.66)	.25	53.3 (9.93)	.25
Anxiety symptoms	57.4 (10.17)	55.5 (9.30)	.19	54.1 (9.93)	.32
Anger	53.5 (9.89)	51.1 (10.42)	.24	50.0 (10.50)	.35
Fatigue	59.7 (8.52)	57.6 (9.46)	.25	55.5 (9.98)	.49
Sleep disturbance	55.6 (9.66)	53.8 (9.40)	.19	54.3 (9.39)	.14
Physical function	40.3 (5.93)	41.2 (7.69)	−.15	41.5 (7.11)	−.20
Social isolation	52.4 (10.53)	50.5 (10.58)	.18	48.9 (11.86)	.33

Physical function coded in reverse, with higher scores corresponding to better physical function. Effect size d calculated as baseline minus follow-up divided by baseline SD. Positive d values indicate improvements, except for Physical function, where negative d values indicate improvement.

Pain interference also showed a significant decrease across the study period (*B* = −.86, *p *< .001). At 4 weeks, 21 out of 68 participants (30.9%) reported at least 30% reductions in pain interference, 5 of whom (7.4%) reported 50% or greater improvements in pain interference. At 12 weeks, 25 out of 70 participants (35.7%) reported reductions in pain interference of at least 30%, 8 of whom (11.4%) reported 50% or greater reductions in pain interference. Effect size calculations indicated medium-to-large magnitude reductions in pain interference from baseline to 4 weeks and from baseline to 12 weeks.

Secondary outcomes showed a similar pattern, with significant improvements in anxiety (*B* = −.86, *p *< .001), depressive symptoms (*B* = −.56, *p *= .001), pain catastrophizing (*B* = −1.63, *p *< .001), fatigue (*B* = −.97, *p *< .001), anger (*B* = −.71, *p *< .001), social isolation (*B* = −.75, *p *< .001), and physical function (*B* = .29, *p *= .013). No significant changes in sleep disturbance were noted across the study period (*B *= −.30, *p *= .082). As shown in Table 3, effect sizes for these secondary outcomes at 4-week follow-up were generally small in magnitude, except for medium effects on pain catastrophizing from baseline to 4 and from baseline to 12 weeks, and on depressive symptoms from baseline to 12 weeks. In general, effect sizes were larger at 12-week follow-up than 4-week follow-up.

### Predictors of treatment response

**Figure 1:**
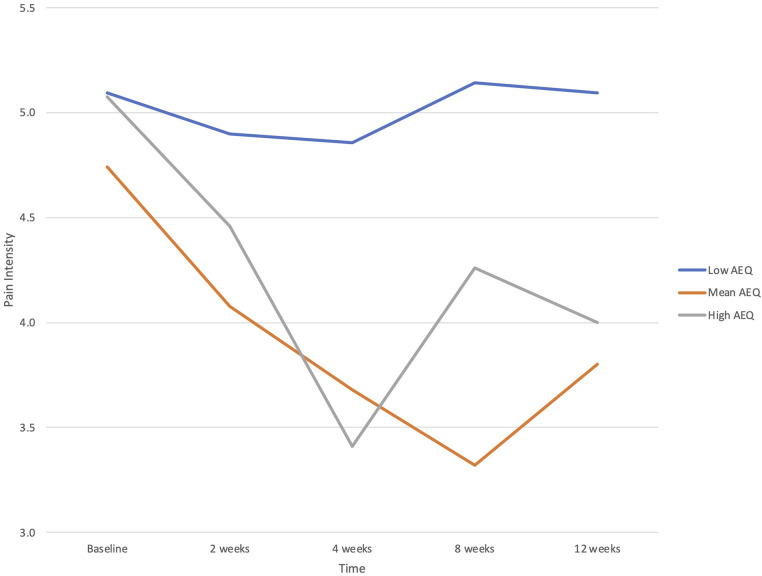
Baseline Ambivalence over Emotional Expression Questionnaire (AEQ) score as a predictor of changes in pain intensity from baseline (before the PSE class) through multiple follow-up assessments to 12 weeks. Plotted are low (−1 SD), mean, and high (+1 SD) values of AEQ.

When potential baseline predictors of treatment response were examined for primary study outcomes, there was a significant interaction between AEQ scores and time in the prediction of pain intensity (*B *= −.170, *p *= .006), but not interference. Participants with higher baseline levels of ambivalence about emotional expression showed greater reductions in pain intensity across time (see Figure 1). Neither ACE scores nor EAC scores predicted treatment responses in pain intensity or pain interference.

**Figure 2:**
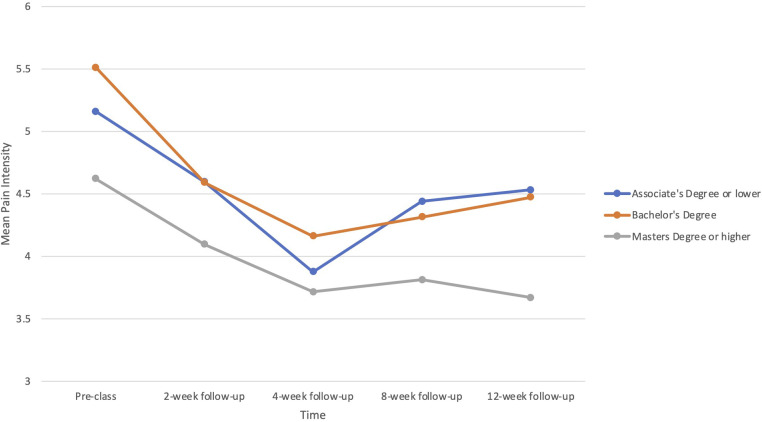
Baseline education level as a predictor of changes in pain intensity from baseline (before the PSE class) through multiple follow-up assessments to 12 weeks.

There was a significant interaction between education and time in the prediction of pain intensity (*B *= −.084, *p *= .036), but not interference. Participants with higher education reported greater reductions in pain intensity, particularly at the 12-week point (see Figure 2). Neither pain duration nor disability status predicted treatment responses in pain intensity or pain interference.

### Class satisfaction ratings

Of the 68 participants who completed class satisfaction ratings, feedback indicated generally positive responses to the class, with mean scores on the 0 to 10 scale ranging from 7.41 (“How likely are you to implement the learnings from this class) to 8.26 (“How well was the experience of chronic pain explained during your class?”). Item-level means, standard deviations, and percentage of respondents rating 8 or higher (i.e., strong endorsement) as well as 2 or lower (i.e., strong disagreement) are presented in Table 4. Between half and two-thirds of the participants strongly endorsed the various satisfaction and utility items about the class, whereas only 2%–10% strongly did not.

**Table 4:** Treatment satisfaction ratings.

Item	M (SD)	Frequency of 8/10 or higher ratings *N* (%)	Frequency of 2/10 or lower ratings *N* (%)
How well was the experience of chronic pain explained during your class?	8.26 (1.79)	47 (69.12)	0 (0)
Did you agree with the type of treatment approaches and recommendations that you received during your class?	7.47 (2.32)	34 (50.00)	2 (2.94)
How satisfied were you with the class?	7.74 (2.24)	40 (58.82)	3 (4.41)
Rate your likelihood to recommend this class to another person who has chronic pain.	7.94 (2.15)	42 (61.76)	2 (2.94)
How relevant was this class to you?	7.46 (2.65)	40 (58.82)	5 (7.35)
How useful was the information presented in this class?	7.50 (2.57)	41 (60.29)	5 (7.35)
How likely are you to implement the learnings from this class?	7.41 (2.76)	42 (61.76)	7 (10.29)

*n* = 68 for all items.

### Sample size and power analysis

The targeted sample size for this uncontrolled study was based on the samples used in other studies of EAET (9, 43) as well as a trial of single-session, CBT intervention (44), which had between 70 and 80 patients. This was assumed to provide adequate statistical power to conduct the primary comparisons (change in primary outcomes from baseline to 4-week and 12-week follow-ups using paired sample *t-*tests). We also conducted a post-hoc power analysis to determine the actual power observed in this study. We found that the sample was adequately powered to detect the smallest observed change among primary outcomes (pain intensity changes from baseline to 12-week follow up; *d *= .42), with a calculated power of.96. All other comparisons involving primary outcomes were powered at.99 or above.

## Discussion

We developed a novel, remote-delivered, group-based, single-session (2-hour) EAET intervention—the “Pain, Stress, and Emotions” class—and tested its clinical effects as well as acceptability and feasibility among people with mixed etiology chronic pain. We delivered this class to four cohorts of patients and assessed changes in clinical measures over 12 weeks, and we tested several baseline measures as predictors of response. Results suggest that this class is both feasible and acceptable, that it results in clinically meaningful change in pain intensity and interference, and that outcomes may be predicted by baseline variables.

With respect to preliminary efficacy of the PSE class, participants showed medium magnitude reductions in pain intensity and near-large magnitude reductions in pain interference over the 12 weeks following the intervention. At the 4-week primary endpoint, one-quarter of participants evidenced reductions in pain intensity of at least 30%, and 13% evidenced substantial pain reductions—50% or greater. Similarly, for pain interference, 31% of participants evidenced clinical reductions of at least 30%, and 7% had reductions of 50% or greater. At the secondary endpoint of 12-week follow-up, the prevalence of reaching 30% reduction in both measures slightly increased. Overall, these results suggest that there are clinically meaningful benefits, at least for some patients, of a brief, remotely delivered class that addresses the role of the brain, stress, and emotions in one's chronic pain, and the possibility of reducing pain by increasing emotional awareness, disclosure, and expression of avoided or suppressed emotions related to stressors, traumas, and conflicts. These results add to the growing evidence base that EAET interventions can lead to clinically meaningful reductions in pain intensity and interference (5, 10–12).

In addition to these benefits for the primary outcomes of pain intensity and interference, the PSE class also led to improvements in most secondary outcomes at 4 weeks: pain catastrophizing, anxiety, depressive symptoms, fatigue, social isolation, anger, and physical functioning. The magnitudes of these changes were somewhat smaller than for primary outcomes, ranging from small to medium, but notably, the effects tended to increase in magnitude at the 12-week point. Other research has found that EAET's effects on secondary outcomes, especially psychological state (anxiety, depression), are smaller and occur later than the effect on pain intensity and interference (5). This pattern may occur because these outcomes are not specifically targeted by EAET, or perhaps because EAET can be emotionally difficult, temporarily increasing one's awareness and experience of negative emotions while reducing the experience of pain. One secondary outcome of note is pain catastrophizing, which had medium to large reductions following the PSE class. A reduction on the Pain Catastrophizing Scale of 7.0 points (as we found at 12-week follow-up) exceeded the clinically meaningful threshold of 6.8 reported in the literature (45). Our findings on pain catastrophizing are notable and aligned with the literature suggesting that pain catastrophizing is highly responsive to behavioral treatments (1, 46, 47) including single-session intervention (48, 49), even when treatments are not specifically designed to reduce pain catastrophizing.

The data indicate that only some patients benefitted from this PSE class, suggesting the importance of identifying predictors of response. We examined several baseline individual difference variables, two of which predicted outcomes. We explored several demographic/pain history variables and found that pain duration and disability status were not predictive, but education was. More educated patients had greater pain reductions, which suggests that greater education, or variables associated with it, facilitates patients' understanding and implementation of the model. We also hypothesized about several variables, and consistent with our hypothesis, patients who reported higher levels of ambivalence over expression of their emotions benefited more from this class—they had greater reductions in pain intensity—than did those with lower baseline ambivalence over emotional expression. Emotional ambivalence is defined as the ongoing internal conflict about the desire to hide emotions despite external circumstances that demand disclosure, and/or regret over decisions to disclose feelings. A key target of EAET and this PSE class is to encourage the expression of emotions that are typically suppressed, with the goal reducing emotional activation and attenuating fears that otherwise trigger and maintain the “pain-danger alarm.” For example, one patient in the class noted that her shoulder pain dropped “from 7 to 0” during the class as she became aware of and gave voice *via* writing and subsequent sharing with other class members her angry feelings toward a relative who had mistreated her, but toward whom she was reluctant to express anything. More generally, the failure to express adaptive “primary” emotions has been linked to prolonged stress responses as well as increased pain (4, 8, 50) suggesting the value of this class particularly for people who fear such disclosure and expression. Two other baseline measures did not predict outcomes, however. Emotional approach coping reflects a positive capacity for emotional awareness and expression rather than a deficit or fear, and it is possible that fear-motivated avoidance (i.e., ambivalence) is more easily addressed by EAET than is an adaptive capacity such as emotional approach coping. We were somewhat surprised that adverse childhood experiences did not predict response to the PSE class, given that adversity / trauma is a key target of EAET. Yet, reporting exposure to childhood adversity does not indicate whether or not these adverse experiences have been resolved or remain problematic, and adulthood or more recent stressors or adversities may be more important for many people's pain than are childhood adversities. Moreover, many people have ongoing psychological or interpersonal conflicts that might activate the pain-danger alarm but are not considered major external events or adversities. Future research should conduct a more detailed and sophisticated assessment of trauma, adversity, and conflict and examine what is being targeted by EAET for each patient.

The PSE class was well received by participants. Nearly two-thirds of the participants reported very high overall satisfaction with the class as well as with the usefulness and relevance of presented information, the likelihood of recommending the class, and use the skills and information learned. These findings suggest that many people with chronic pain are interested in the role of stress and emotions and find a class addressing these topics to be of value. It should be noted, however, that a small minority of patients (less than 10%) found the class to be of little or no value. Comments from such patients suggest that they did not view the model as applicable to their unique type of pain, or they were generally skeptical of a model linking stress and emotions to pain. Overall, however, participant feedback was aligned with therapist observations that the class was positively received by most participants and strongly positively impacted a subset of them, who often reported newfound awareness and meaningful reductions in pain intensity and interference.

Although a growing body of research exists on the efficacy of EAET for chronic pain management conditions (5, 9, 11, 12, 19, 20, 51), the use of a telehealth platform to deliver interventions for chronic pain—particularly single-session interventions—remains novel and understudied. This study demonstrates the feasibility and effectiveness of a single-session version of EAET that is delivered remotely and to groups of patients. It shows that, despite the expectation that patients would engage in emotionally challenging experiences (disclose adversities, express avoided emotions, etc.), patients were accepting and engaged. These observations challenge potential skepticism by clinicians and scholars that an EAET course may not be well received or that it would have feasibility problems when conducted in a group setting or tele-health format. These results also broadly highlight the public interest in the online delivery of interventions as a home-based chronic pain treatment modality. Overall, this study extends prior work (52–59) supporting the utility, user satisfaction, and efficacy of videoconference-delivered interventions for chronic pain.

### Study limitations and future directions

The main limitation of this study is the uncontrolled design. The lack of a randomized control condition precludes making causal statements about this PSE class. For example, it is possible that there was some natural improvement or even statistical regression to the mean led to some of the observed improvements in outcomes. However, such effects are likely to be relatively small given the fact that we invited people from a list of research volunteers independent of their current pain or distress, rather than soliciting patients from the community who often participate in research when they are experiencing elevated pain or distress, which often remits substantially over the ensuing weeks and months. Also, the lack of an active control condition—such as one that meets for an equivalent duration and engages in education of another topic—precludes conclusions that something specific about this PSE class was responsible for the improvements. studies need to include control conditions such as waitlist/treatment-as-usual and an active control condition. A direct comparison of this PSE class with a bonafide alternative model such as a CBT-based pain management class would also be of value to determine whether the new model is more powerful than a traditional model, and to explore who might benefit from each approach.

A second limitation of our study is self-selection, which could bias study findings or limit generalization only to those who are, for example, motivated to explore the role of stress in their pain. Nonetheless, potential participants were not provided much detail about the content of the intervention, as it was described only as a “pain psychology class,” and recruitment efforts targeted patients who agreed to be contacted for research purposes after seeking care at a tertiary pain clinic. Future studies should examine specific motivation factors and treatment expectancies as predictors of treatment response.

A third limitation of our study is that the sample was quite heterogeneous with respect to types of chronic pain; most participants reported multiple pain diagnoses, and a few endorsed up 8 or more diagnoses. EAET is designed specifically for nociplastic or primary pain, for which there appears to be a larger role of stress and emotional factors than is found in secondary (nociceptive or neuropathic) pain (60). Thus, it is impressive that the current study found the substantial effects and satisfaction that it did, given that it included some people with these latter pain types (e.g., arthritis, trigeminal neuralgia, neuropathic pain). Future research with larger samples should examine type of pain as a predictor of PSE outcomes or limit the patient sample to those with primary/nociplastic pain, where even larger effects would be expected.

Fourth, although this trial had minimal exclusions for participation, increasing its generalizability, the sample was biased toward higher education and socioeconomic status and under-representative of people of color and those with serious pain-related disability; more diverse populations need to be studied. Finally, although the sample had sufficient size to provide powerful tests of within-subject effects over time, larger samples would provide not only more reliable estimates of effect size but permit more robust tests of predictors of intervention effects.

## Conclusions

Despite these limitations, this study found high satisfaction and evidence of clinically meaningful benefits of this single-session, remotely delivered class based on EAET. Our findings provide initial evidence that PSE's online delivery may efficiently reduce the burden of chronic pain and improve symptom management. The findings are particularly promising given that the class is delivered in a single session, and participants have no ongoing therapist contact. In addition, the COVID-19 context and the online delivery modality support the ecological validity of the study findings, and this class addresses the rapidly expanding need for alternatives to face-to-face encounters. Online delivery of PSE stands to address pain disparities by ensuring rapid and more equitable access to pain treatment, and because it is readily extendable to underserved populations (e.g., rural locations) and may be offered at a low cost. Although this telehealth class is likely not appropriate for all people with chronic pain, and many patients will need additional treatment to make meaningful changes in their stress, emotions, and pain, this PSE class appears to provide a valuable start.

## Data Availability

The raw data supporting the conclusions of this article will be made available by the authors upon request, without undue reservation.
